# Structure of the C-terminal domain of the surface antigen SpaP from the caries pathogen *Streptococcus mutans*

**DOI:** 10.1107/S174430911004443X

**Published:** 2010-12-21

**Authors:** Åsa Nylander, Nina Forsgren, Karina Persson

**Affiliations:** a Department of Odontology, Umeå University, SE-901 87 Umeå, Sweden

**Keywords:** antigen I/II, isopeptide bonds, dental plaque

## Abstract

SpaP is a 1500-residue adhesin expressed on the surface of the caries-implicated bacterium *Streptococcus mutans*. SpaP is a member of the antigen I/II (AgI/II) family of proteins expressed by oral streptococci. These surface proteins are crucial for the incorporation of streptococci into dental plaque. The structure of the C-terminal domain of SpaP (residues 1136–1489) was solved and refined to 2.2 Å resolution with six molecules in the asymmetric unit. Similar to a related AgI/II structure, SpaP is stabilized by isopeptide bonds between lysine and asparagine side chains.

## Abstract 2

The structure of the C-terminal domain of the *S. mutans* surface adhesin SpaP has been determined to 2.2 Å resolution.

## 1. Introduction

*Streptococcus mutans* is the causative agent of caries (Loesche, 1986 ▶) and its surface adhesin SpaP is important for its incorporation into the oral biofilm (dental plaque). SpaP is a member of the antigen I/II (AgI/II) family of proteins that are expressed on the surfaces of oral streptococci and are key molecules for networking with other microorganisms and host cells in the oral biofilm. AgI/II proteins are approximately 1500 amino acids in length and consist of seven regions: a leader peptide (∼38 amino acids), an N-terminal region (∼80 amino acids), an alanine-rich region (∼320 amino acids), a variable domain (∼360 amino acids), a proline-rich region (∼180 amino acids), a C-terminal domain (∼500 amino acids) and a cell-wall-anchoring segment (Jenkinson & Demuth, 1997 ▶). The structure of AgI/II proteins is unique in the sense that the proline-rich region and the alanine-rich region wrap around each other, forming an elongated fibrillar structure that presents the variable domain at the tip (Larson *et al.*, 2010 ▶). The structure further consists of a C-terminal domain which contains an LP*X*TG motif that anchors the protein to the cell wall. A crystal structure of the major part of the C-terminal domain of the AgI/II protein SspB from the commensal bacterium *S. gordonii* (Forsgren *et al.*, 2010 ▶) showed that the protein consists of two structurally similar β-sandwich domains. Sequence analysis indicates that the C-terminal region also contains a third β-sandwich domain. It has been shown that the C-terminal domain of *S. gordonii* SspB, but not *S. mutans* SpaP, functions as an adherence site for the oral pathogen *Porphyromonas gingivalis*, which is associated with adult perio­dontitis, a condition that may result in tooth loss. In order to compare the structure of *S. mutans* SpaP with the previously determined structure of SspB from *S. gordonii*, we determined the crystal structure of SpaP-C_1136–1489_, which is reported here.

## 2. Materials and methods

### 2.1. Cloning

The preparation of chromosomal DNA from *S. mutans* SK773 was performed using BacReady from GenScript. Constructs were designed based on the *spaP* gene from *S. mutans* strain NG5 (accession code X17390) and the domain borders that were used to solve the analogous structure SspB from *S. gordonii* (Forsgren *et al.*, 2010 ▶). The fragment was PCR-amplified using the forward primer 5′-AAAAA**CCATGG**AAGCTAAATTAGCAAAA-3′ and the reverse primer 5′-­TTTTT**GGTACC**TTATGGAGTTTTTACCGGTGGAGT-3′ (restriction sites are shown in bold). The PCR product was digested with *Nco*I and *Acc*65I and ligated into the equivalent sites of the pET-M11 expression vector (kindly provided by Gunter Stier, EMBL, Germany) containing a His tag at the N-terminus. The final construct encodes His_6_-PMSDYDIPTTENLYFQGAM-SpaP_1136–1489_ with a molecular weight of 44.1 kDa. The plasmid was transformed into *Escherichia coli* DH5α and colonies were subsequently selected on kanamycin plates. The positive clones were verified by DNA sequencing.

### 2.2. Overexpression and purification

The SpaP-C_1136–1489_ construct was overexpressed in *E. coli* BL21 pLysS cells at 310 K in Luria broth supplemented with 50 µg ml^−1^ kanamycin and 25 µg ml^−1^ chloramphenicol. When the culture reached an OD_600_ of 0.6, the temperature was lowered to 298 K, protein expression was induced with 0.5 m*M* IPTG and the culture was allowed to grow for 4 h. The cells were harvested by centrifugation at 5300*g* for 20 min and the cell pellets were stored at 193 K. The cell pellets were resuspended in 20 m*M* Tris pH 7.5, 150 m*M* NaCl and 10 m*M* imidazole pH 8.0 supplemented with EDTA-free protease-inhibitor cocktail (Roche). The cells were lysed on ice by sonication and cellular debris was removed by centrifugation at 39 000*g* for 50 min. The supernatant was loaded onto an Ni–NTA agarose column (Qiagen). The SpaP-C_1136–1489_ protein was washed with 20 m*M* Tris pH 7.5, 150 m*M* NaCl and 20 m*M* imidazole pH 8.0 and eluted in 20 m*M* Tris pH 7.5, 150 m*M* NaCl and 300 m*M* imidazole pH 8.0. The buffer was exchanged to 20 m*M* Tris pH 7.5, 150 m*M* NaCl and 0.5 m*M* EDTA and the protein was further purified by size-exclusion chromatography on a Superdex 200 16/60 PG column (Amersham Bio­sciences). The protein purity was analyzed by SDS–PAGE (data not shown).

### 2.3. Crystallization

The SpaP-C_1136–1489_ protein was concentrated to 15 mg ml^−1^ in 0.2 m*M* Tris pH 7.5 using an Amicon Ultra centrifugal filter device (Millipore). Initial crystallization trials were performed with a Mosquito pipetting robot using sitting-drop vapour diffusion and standard crystal screening kits (Hampton Research and Molecular Dimensions) in 96-well plate format. The initial hits were optimized using the hanging-drop method by mixing 2 µl protein solution at a concentration of 15 mg ml^−1^ and 1 µl reservoir solution, resulting in crystals that were suitable for data collection. The final crystallization condition was 0.1 *M* sodium acetate pH 5.0 and 15%(*w*/*v*) polyethylene glycol 6000.

### 2.4. Data collection and processing

Prior to data collection, the crystals were soaked in crystallization solution supplemented with 20% polyethylene glycol 400, mounted on loops and vitrified in liquid nitrogen. Diffraction data were collected to 2.2 Å resolution at 100 K using a MAR CCD 165 detector on beamline I911-­5, MAX-lab, Lund, Sweden. The diffraction data were processed with *XDS* (Kabsch, 2010 ▶) and scaled with *SCALA* from the *CCP*4 program suite (Collaborative Computational Project, Number 4, 1994 ▶).

### 2.5. Structure solution and refinement

The initial molecular-replacement search model was generated from *S. gordonii* SspB-C (PDB code 2woy; Forsgren *et al.*, 2010 ▶) and the *S. mutans* SpaP protein sequence using the program *CHAINSAW* (Stein, 2008 ▶) The sequence identity between the two proteins is 67% in this region. The structure was solved using the program *Phaser* (McCoy *et al.*, 2007 ▶) and was refined with *phenix.refine* (Afonine *et al.*, 2005 ▶). The first refinement steps included rigid-body refinement and simulated annealing starting at 5000 K; translation–liberation–screw (TLS) refinement was used in the last rounds of refinement, treating each chain as an individual TLS group (Winn *et al.*, 2001 ▶). Manual inspection, rebuilding and addition of water molecules were performed with *Coot* (Emsley *et al.*, 2010 ▶). The quality of the model was analyzed with *WHAT_CHECK* (Hooft *et al.*, 1996 ▶). The model was subjected to sixfold NCS restraints throughout refinement. Refinement statistics are given in Table 1 ▶. Figures were drawn with *CCP*4*MG* (Potterton *et al.*, 2004 ▶)

Structure factors and coordinates have been deposited in the Protein Data Bank (PDB; http://www.rcsb.org/pdb) under accession code 3opu.

## 3. Results and discussion

### 3.1. Crystallization, data collection and structure determination

Crystals of the SpaP-C_1136–1489_ protein were obtained by hanging-drop vapour diffusion. The crystals belonged to space group *P*2_1_2_1_2, with six molecules in the asymmetric unit, which corresponds to a Matthews coefficient of 2.68 Å^3^ Da^−1^ (54% solvent content; Matthews, 1968 ▶). The unit-cell parameters were *a* = 135.8, *b* = 238.4, *c* = 78.5 Å. The structure was solved by molecular replacement using a model based on the analogous *S. gordonii* structure as a search model. Refinement and manual fitting of the model resulted in a final *R*_work_ of 21.0% and an *R*_free_ of 24.8%.

### 3.2. The overall structure of SpaP-C_1136–1489_

**Figure 1:**
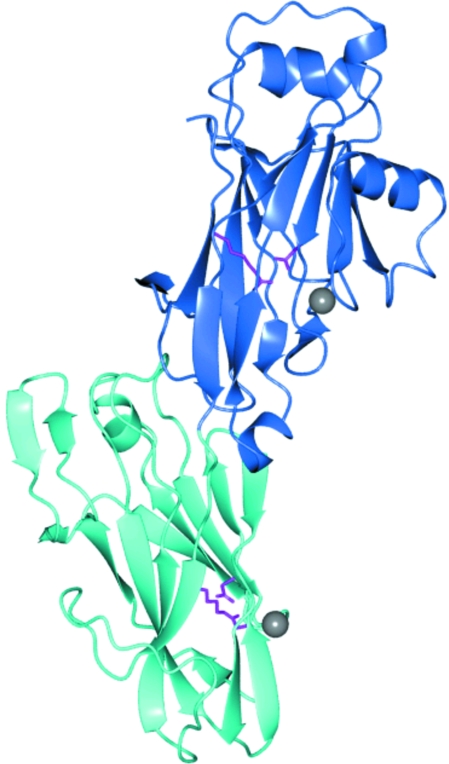
The overall structure of SpaP-C_1136–1489_. The C2 domain is shown in blue and the C3 domain is shown in light blue. Two calcium ions are depicted as grey spheres and the two isopeptide bonds are shown as stick models in pink.

The final model of SpaP-C_1136–1489_ from *S. mutans* consists of six independent protein chains, each binding to two Ca^2+^ ions. 545 water molecules were included in the model. The refined monomers consist of residues 1152–1489. The 16 N-terminal residues and residues from the linker could not be seen in the electron density and were not included. The crystallized protein consists of two domains, C2 and C3, each of which adopts a β-sandwich fold (Fig. 1 ▶). The C2 domain consists of two β-sheets with five and six strands. The domain also contains two short α-helices located on top of the β-­sandwich, as well as one helix positioned perpendicular to the β-­sandwich. The C3 domain consists of two sheets built up of five strands each. On the side facing the C2 domain the β-sandwich strands are connected by long loops, which also include a short helix and a short β-hairpin. On the other side the strands are connected by short turns, with one exception in which a long coil (16 amino acids) connects two strands. Each domain is stabilized by a metal ion, modelled as Ca^2+^. In each β-­sandwich the two sheets are linked by a covalent isopeptide bond formed between the side chains of a lysine and an asparagine.

The six monomers in the asymmetric unit are generally very similar, with a root-mean-square deviation (r.m.s.d.) ranging from 0.3 to 0.7 calculated on all C^α^ atoms.

### 3.3. Isopeptide bonds and Ca^2+^ sites

In the C2 β-sandwich an isopeptide bond is formed between Lys1157 of one sheet and Asn1307 of the other. Similarly, an iso­peptide bond is formed between Lys1334 and Asn1469 of the C3 β-­sandwich. Both isopeptide bonds are formed between the NZ atom of the lysine and the CG atom of the asparagine, thereby releasing ammonium. A conserved aspartic acid forms hydrogen bonds to the peptide C=O and NH groups. The isopeptides are further surrounded by hydrophobic and aromatic residues. The hydrophobic environment is more prominent in the C3 domain. The isopeptide bonds and their surrounding residues are very similar to what was reported previously for the analogous protein SspB from *S. gordonii* (Forsgren *et al.*, 2010 ▶). Isopeptides are being described in an increasing number of Gram-positive surface proteins, for example in the pili from *S. pyogenes* and *Corynebacterium diphtheriae* (Kang *et al.*, 2007 ▶, 2009 ▶). It is hypothesized that these covalent bonds are used as a means of stabilizing these surface proteins against mechanical force.

The Ca^2+^ ions are likely to play important structural roles since they are located in close proximity to the isopeptide bond in each domain. In both domains the aspartic acid that forms hydrogen bonds to the isopeptide is followed by an aspartic acid that coordinates a Ca^2+^ ion. The Ca^2+^ ion in the C2 domain is coordinated by carboxylate groups from two residues, Asp1208 and Glu1211, the carbonyl O atoms of Lys1261, Tyr1209 and Ala1263 and one water molecule. In C3 the Ca^2+^ ion is coordinated by side-chain O atoms from Glu1387 and Asp1384, the main-chain O atoms of Lys1430, Gly1431 and Tyr1385 and one water molecule.

### 3.4. Comparison with the *S. gordonii* SspB structure

**Figure 2:**
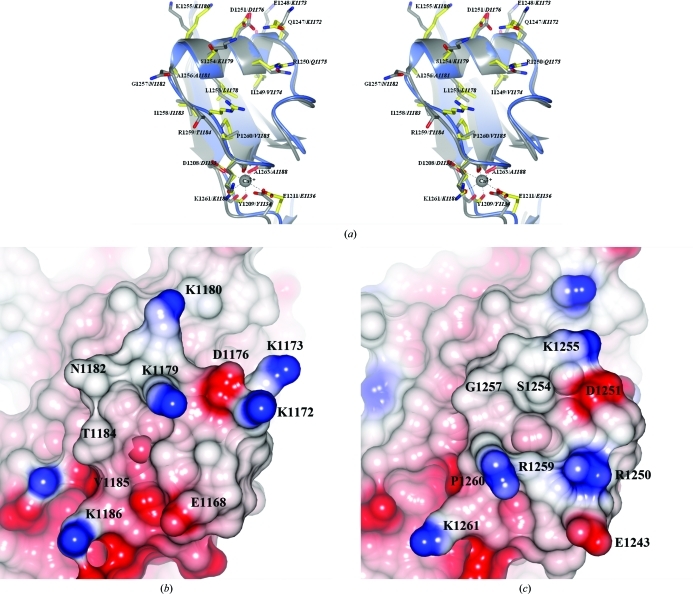
Comparison of the SspB and SpaP BAR regions. (*a*) The BAR motif in the *S. gordonii* AgI/II protein SspB is the recognition site for *P. gingivalis* and consists of a helix (KKVQDLLKK) followed by an extended region (NITVK). A calcium ion (grey sphere) stabilizes the motif through interactions (broken lines) with three main chains, two side chains and a water molecule. The analogous region in *S. mutans* SpaP is composed of an α-helix (QEIRDVLSKA) followed by an extended region (GIRPK) which share only 40% sequence identity with *S. gordonii* SspB. The two proteins are superimposed and shown in stereo. SspB is shown in grey and SpaP in blue and yellow. The residues are labelled, with the SspB residues in italics. (*b, c*) Electrostatic surface comparison of the BAR regions in SspB and SpaP. The SspB BAR region (*b*) and the equivalent region in SpaP (*c*) generate very different electrostatic surfaces and thus a different recognition pattern.

The overall structure of *S. mutans* SpaP-C is generally very similar to the structure of *S. gordonii* SspB-C, with the isopeptide bonds and Ca^2+^ atoms in identical positions. The approximate r.m.s.d. between the two structures is 1.0 Å. The largest deviation in sequence is found in the helix located perpendicular to the β-sandwich of the C2 domain and the following extended region. This region is described as the BAR region (SspB adherence region) in *S. gordonii* SspB and is used as a recognition handle by the periodontal pathogen *P. gingivalis* (Brooks *et al.*, 1997 ▶). This region of SpaP is not recognized by *P. gingivalis* despite its very similar structure (Fig. 2 ▶ *a*). Most of the charged residues in the helix facing the solvent are different in the two proteins (QEIRDVLSK in SpaP and KKVQDLLKK in SspB), creating different conditions for recognition. The following extended regions are also dissimilar in sequence (GIRPK in SpaP and NITVK in SspB). Previous studies have shown that two single mutations, N1182G and V1185P, in the extended region of SspB that make the region more like that of the *S. mutans* SpaP protein completely abolish *P. gingivalis* adherence (Demuth *et al.*, 2001 ▶). The crystal structure of SpaP shows that the natural occurrence of these amino acids in the protein does not alter the secondary structure of the region and gives limited clarification of the importance of these two single sites with regard to *P. gingivalis* recognition and adherence. However, the electrostatic surfaces are considerably different (Figs. 2 ▶ *b* and 2 ▶ *c*) and create very different recognition properties for any interaction partner. Comparison of the structures of SpaP and SspB opens the possibility of developing substances to eliminate the adherence of *P. gingivalis* to the oral biofilm.

## Supplementary Material

PDB reference: C-terminal domain of SpaP, 3opu

## Figures and Tables

**Table 1:** Data-collection and processing statistics Values in parentheses are for the outer shell.

Wavelength (Å)	0.90852
Space group	*P*2_1_2_1_2
Unit-cell parameters (Å)	*a* = 135.8, *b* = 238.4, *c* = 78.5
Molecules in asymmetric unit	6
Resolution range (Å)	29.50–2.18 (2.30–2.18)
No. of observed reflections	979090
No. of unique reflections	132886
Wilson *B* factor (Å^2^)	27.7
Completeness (%)	99.7 (98.8)
Multiplicity	7.4 (7.0)
〈*I*/σ(*I*)〉	28.5 (12.2)
*R*_merge_†	0.049 (0.170)
*R*_work_‡	0.210
*R*_free_‡	0.248
R.m.s.d. bonds (Å)	0.010
R.m.s.d. angles (°)	1.228
Overall *B* factor (Å^2^)	
Protein	25.8
Water	23.3
Calcium	33.6
Ramachandran statistics (%)	
Preferred regions	95.24
Allowed regions	3.72
Outliers	1.04
PDB code	3opu

† *R*_merge_ = 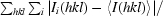 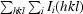, where *I*_*i*_(*hkl*) is the intensity of the *i*th observation of reflection *hkl* and 〈*I*(*hkl*)〉 is the average over all observations of reflection* hkl.*

‡ *R*_work_ = 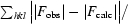 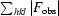, where *F*_obs_ and *F*_calc_ are the observed and calculated structure-factor amplitudes, respectively. *R*_free_ is *R*_work_ calculated using 5% of the data that were omitted from refinement.
